# Adapting a Parental Support App to Promote Healthy Diet and Physical Activity Behaviors (MINISTOP) for a Multi-Ethnic Setting: A Qualitative Study on the Needs and Preferences of Parents and Nurses within Swedish Child Health Care

**DOI:** 10.3390/nu13072190

**Published:** 2021-06-25

**Authors:** Christina Alexandrou, Ulrika Müssener, Kristin Thomas, Hanna Henriksson, Marie Löf

**Affiliations:** 1 Department of Health, Medicine and Caring Sciences, Division of Society and Health, Linköping University, 581 83 Linköping, Sweden; ulrika.mussener@liu.se (U.M.); kristin.thomas@liu.se (K.T.); hanna.henriksson@liu.se (H.H.); marie.lof@liu.se (M.L.); 2 Department of Biosciences and Nutrition, Karolinska Institute, NEO, Group MLÖ, 141 83 Huddinge, Sweden

**Keywords:** preschool, parental support, multi-ethnic, immigrant, health behaviors, child health care nurses, healthy diet, physical activity, qualitative methods, mHealth app

## Abstract

Early efforts for prevention of childhood overweight and obesity are needed. In order to adapt an app promoting healthy diet and physical activity behaviors in children (MINISTOP 1.0) for multi-ethnic communities, we explored: (1) needs and concerns among Somali-, Arabic-, and Swedish-speaking parents in terms of supporting healthy diet and activity behaviors in their children; (2) nurses’ perceptions of parental needs and concerns in relation to diet and physical activity behaviors; and (3) how the features and content of the MINISTOP 1.0 app could be refined to better support health behaviors in children, among both parents and nurses. Focus groups with Somali-, Arabic-, and Swedish-speaking parents (*n* = 15), and individual interviews with nurses (*n* = 15) were conducted. Parents expressed several challenges in supporting children’s health behaviors, the need for a tailored app, and alternative ways of accessing the content (audio/video). Nurses emphasized the need of supporting parents early, and the value of a shared platform in different languages, to facilitate communication. This study contributes valuable insights about parental needs and relevant adaptations to a parental support app, such as addition of audio/video files for increased accessibility. This adapted app version—MINISTOP 2.0, can be useful for childhood obesity prevention in multi-ethnic communities.

## 1. Introduction

Childhood obesity is a health challenge worldwide; in 2019, an estimated 38 million children under the age of five were overweight or obese [1,2]. In Sweden, around 10–15% of four-year-old children are either overweight or obese [3,4], and recent data points towards a continuous increase in obesity rates among school-aged children [5]. Preventive efforts to counteract this development have been called for already in the preschool age (2–5 years) [6], with primary child health care nurses being key players for delivering interventions [7]. Nurses within Swedish primary child health care have a unique role since they regularly meet more than 99% of children in Sweden aged 0–5 years [8]. This allows them to follow each child’s development and work preventively, as well as to support families with different needs [8]. However, thus far, effective and scalable interventions to counteract obesity for this setting in Sweden and other countries are largely lacking.

Migration continues to increase worldwide, and many countries are thus becoming multi-ethnic and culturally diverse [9]. To date, 25% of all children in Sweden have a foreign background [10], with the largest ethnic groups being from Syria, Somalia, and Iraq [11]. Hence, adaptations are central in order to enable interventions to become accessible and inclusive for Somali- and Arabic-speaking families in Sweden. This is specifically important considering the socioeconomic gradient of obesity observed in high-income countries [12,13]. The prevalence of obesity is, for instance, twice as high among children living in socioeconomically disadvantaged areas in Sweden [14]. In this context, it is relevant to note that previous research from other countries has indicated that when migrating to a new country, parents often struggle with conflicting information and advice on infant and child feeding practices from their traditional family and cultural networks and the new country’s child health services [15,16]. Furthermore, Arcan et al. found that Somali immigrant parents in the US feel challenged by their own uncertainties about healthy diet and activity behaviors as well as by their children’s preferences for unhealthy foods [17]. Moreover, Aljunaibi et al. found that parents in the United Arab Emirates often underestimated their child’s weight according to BMI [18], while Almarhoon et al. observed that pressure to eat and restrictive feeding practices were common among Saudi Arabian mothers [19]. However, more knowledge on the views and potential challenges perceived by Somali- and Arabic-speaking families living in Sweden is needed.

Mobile health (mHealth) is a rapidly growing research field that aims to study the effect of mobile phone-based interventions on health and behaviors [20,21]. The advantages of mHealth solutions include less personnel resources and the possibility to access intervention content at any time and wherever the user is [22]. In addition, mHealth interventions provide great flexibility in terms of translation and modifications to provide inclusive content and features. We have previously developed the MINISTOP app, which provides a six-month mHealth program aiming to support parents to promote healthy diet and physical activity behaviors and prevent obesity in preschool-aged children [23]. The app delivers a new comprehensive theme on healthy diet and activity behaviors for preschool-aged children every two weeks, over a period of six months. The app also includes a feature where parents can register and receive weekly feedback on their child’s intake of key dietary items such as fruit and vegetables, candy, and sweetened beverages, as well as time spent being active and screen time. The effectiveness of the app has been evaluated in a randomized controlled trial (Trial Registration: ClinicalTrials.gov NCT02021786) with accurate and objective outcome measures, demonstrating a statistically significant improvement on a composite score of six dietary and physical activity behaviors, especially in children with a higher fat mass index [23]. However, accessibility to the app for parents speaking other languages was limited, as this first version was available only in Swedish. Thus, the next step is to collect relevant information from child health care nurses and parents to develop an app that is appropriate for a multi-ethnic setting. This work should also include Swedish-speaking parents in order to receive perceptions and suggestions for improvements to the Swedish version.

To improve the MINISTOP 1.0 app and facilitate its use in multi-ethnic communities, a qualitative study was performed to explore the preferences of end-users (parents) and child health care nurses regarding a parent-oriented mHealth solution. The perspectives of both parents and nurses were included to get a more robust and comprehensive understanding. Specifically, this study aimed to explore: (1) needs and concerns among Somali, Arabic-, and Swedish-speaking parents in terms of supporting heathy diet and activity behaviors in their children; (2) nurses’ perceptions of parental needs and concerns in relation to diet and physical activity behaviors; and (3) how features and content of the MINISTOP 1.0 app could be refined in order to support healthy diet and activity behaviors in children, among both parents and nurses.

The findings from this study were used to create a second version of the app, MINISTOP 2.0, which is currently under evaluation within the primary health care setting across Sweden using a hybrid Type I effectiveness-implementation design (Trial Registration: ClinicalTrials.gov NCT04147039) [24].

## 2. Materials and Methods

### 2.1. Ethical Considerations

The current study is a part of the MINISTOP 2.0 project [24], which has been approved by the Swedish Ethical Review Authority (Ref No. 2019-02747; 21 August 2019).

### 2.2. Study Design

A qualitative design [25] was used. Data collection was conducted through focus group interviews with parents and individual interviews with child health care nurses. The study followed the Consolidated Criteria for Reporting Qualitative Research (COREQ) checklist (Figure S1) [26].

### 2.3. Setting, Participants and Recruitment

Purposive sampling was conducted at a child health care center in Ryd, a socioeconomically diverse district in Linköping, Sweden, with a predominantly immigrant population. The inclusion criteria were: (1) Somali-, Arabic-, and Swedish-speaking parents, (2) willingness to participate and (3) those who owned a smartphone. Recruitment of Swedish-speaking parents was performed by a child health care nurse, while Somali- and Arabic-speaking parents were recruited by “bridge builders”—professionals with immigrant backgrounds who work as a link between families and the Swedish health care system [27]. Both the nurse and the bridge builders were employed at the child health care center. Recruitment of all parents took place during routine visits to the child health care center in September 2019. Parents were provided with written and verbal information about the study aims and procedure, in their respective language. In total, 16 parents were invited and agreed to participate. One parent invited to the Arabic focus group was not able to attend due to sickness. Thus, three focus group interviews (Somali, *n* = 5; Arabic, *n* = 4; and Swedish, *n* = 6) were conducted in October 2019.

Convenience sampling was conducted for the interviews with the nurses. Recruitment was performed in September 2019 via child health care centers (*n* = 24) that had already agreed to participate in the MINISTOP 2.0 trial [24]. The inclusion criteria were: (1) currently employed at one of the child health care centers and (2) willing to participate. Invitations were sent via email by the first author of the manuscript, a female PhD student and nutritionist (CA). Written information about the study aims and procedure was enclosed. Nurses registered their interest by replying to the email. In total, 15 nurses from nine child health care centers replied to the invitation and an appointment for the interview was scheduled with each nurse. Participants represented child health care centers from various socioeconomic and geographical areas in Sweden.

### 2.4. Data Collection

Semi-structured interview guides [25] were developed within the research group with expertise in nutrition, rehabilitation and behavioral science, including the development and evaluation of mHealth interventions, as well as qualitative methods. The interview guide for the focus groups (see Table S1) aimed to capture parents’ perceived needs of support for promoting healthy diet and activity behaviors in their children, and parents’ preferences regarding useful content and features in a parental support app. Parents also provided feedback on the current content and features of the MINISTOP 1.0 app. The interview guide for the individual interviews (see Table S2) aimed to capture current health promotion practice routines, and conditions for using new mHealth tools in routine practice. Similar to the parents, nurses provided feedback on the MINISTOP 1.0 app. Both parents and nurses were shown enlarged demo screenshots of the app during their interview, so that they would be able to reflect on the content and features of the MINISTOP 1.0 app. All interviews were audio recorded and transcribed verbatim.

### 2.5. Focus Group Interviews

The interviewer was CA, while HH, a dietician and female researcher with a PhD and training and experience in qualitative methodology, took notes, asked follow-up questions and kept track of time. The focus group interviews with the Somali and Arabic speaking parents were conducted together with a translator. Before the start of each focus group, parents were reminded about the interview aims and procedure, signed a consent form and answered some demographic questions about themselves (e.g., age, country of birth, education, number of children). The focus groups were 68 min in duration on average, and ranged from 62 to 78 min.

### 2.6. Individual Interviews

All individual interviews were conducted over the phone by CA. Prior to each phone interview, CA emailed the nurse a PDF document with screenshots of the MINISTOP 1.0 app, to be viewed together during the interview. Informed verbal consent was obtained and recorded at the beginning of each interview. The interviews had an average duration of 61 min and ranged from 37 to 90 min.

### 2.7. Data Analysis

After each interview, notes and comments on initial thoughts and ideas were made by CA. All interviews were thereafter transcribed verbatim by an external transcribing firm. Thematic analysis was used to analyze the data [28]. An inductive approach was chosen to explore the latent meanings and create a deeper understanding of the participants’ perspectives [29]. The analyses followed a prescribed, sequential process where as follows: overall impressions were noted, data were reduced and coded into initial themes, patterns and interconnections were searched for, final themes were mapped and built, and conclusions were drawn. Initially the transcribed data was read by CA and UM, an occupational therapist and a female researcher with a PhD and training and expertise in qualitative methodology.

During the first reading a comprehensive understanding was acquired, and quotations were selected. Preliminary themes from the data emerged through an iterative analysis process of reading and rereading the selected quotations, searching for patterns. Data saturation was reached when no new codes and themes were found [30]. Coding into themes was initiated by the first author and later, discussions between the two researchers led to the formation of four themes. The selected quotations and identified themes were presented and discussed among all authors. Agreement was reached on the quotations to be included, and boundaries for the themes were established jointly.

## 3. Results

Parents’ and nurses’ characteristics are presented in Table 1. All participants, except for one parent in the Swedish focus group, were female. Themes are described for each aim and illustrated with quotations below (see also Table 2). Excerpts from the interview transcripts are presented to support and exemplify the categorization. The number after each quotation represents the interviewee’s id within the group, followed by the number of children for the parental quotations, or by the number of years in the profession for quotations from the nurses. An ellipsis (…) indicates that text has been omitted.

### 3.1. Needs and Concerns

#### 3.1.1. Parental Challenges

The first theme relates to our aim regarding needs and concerns among Somali-, Arabic-, and Swedish-speaking parents. The theme highlights how parenthood is characterized by a series of perceived challenges, relating to the parental role and responsibilities of engaging in healthy living.

Parents in all three focus groups expressed challenges in relation to supporting healthy eating and diet in their children. In the Arabic focus group, one parent mentioned, that despite receiving information about healthy eating from the Swedish health care system, she still felt a need for more extensive information, specifically addressing parental strategies. According to the parent, it would be beneficial to receive such information and support in good time before being faced with a challenging situation.

“Just to be able to tell somebody and reflect upon that ‘Well, but it’s not [just] my child’ (…) that you receive [information] a little earlier than gaining your own experience (…). Should it be like this? Am I doing the right thing?” (Mother No. 4, Arabic focus group, one child)

In the Somali focus group, parents brought up the challenges of handling different food cultures, i.e., the Somali food culture at home, and then the Swedish food culture at their child’s preschool. The main concern was that children did not eat enough during the hours spent at preschool.

“We have different habits, the Swedish and ours [Somali]. For example, we do not eat boiled potatoes at home, but in Sweden you eat them. […]. My children usually come home hungry when they have been served potatoes and such at school, because they do not like that kind of food.” (Mother No. 2, Somali focus group, seven children)

One of the parents in the Somali focus group also talked about how behaviors connected to worrying, e.g., parental pressure to eat instead of allowing children to self-regulate their intake of food, only made children more averse to certain foods. Instead, she highlighted the need for clear and reassuring information about these issues.

In the Swedish focus group, extensive health information was described as a challenge and something that could have a negative influence on the parenting role. For instance, some argued that parents were already stressed in their everyday lives, and that trying to follow recommendations only added to the feeling of not being a good enough parent. Instead, they emphasized the need for information reassuring the parent not to stress about food.

“A bit of encouragement that it is temporary, that you should not stress too much when things are not going the way you would like them to.” (Mother No. 1, Swedish focus group, one child).

Achieving a healthy level of screen time was another challenge expressed among the participants. In the Swedish focus group, parents mentioned children missing out on social interactions when spending too much time in front of a screen. Similarly, parents in the Somali focus group were worried that even very young children could sit for hours in front of a screen if the parents did not intervene. In the Arabic focus group, one parent expressed feelings of resignation regarding her child’s screen time.

“My child does not want to eat, or it takes a long time for her to eat. […] She wants to watch [something on] the phone at the same time [as she eats], not TV, but she only eats if she can watch the phone at the same time.” (Mother No. 3, Arabic focus group, one child).

Parents in the Swedish focus group had no particular concerns regarding their child’s physical activity; they believed that children engaged in more than enough physical activity at preschool and therefore going out to play in the evening was not prioritized. In the Arabic and Somali focus groups parents talked about how the weather was often a barrier for them. However, they were curious about what was recommended for outdoor play. For the Somali parents, practical reasons such as having many children to dress was an additional barrier to outdoor play, especially during the colder seasons.

#### 3.1.2. Supporting Parents

The second theme refers to our second aim regarding needs and concerns from a nurse’s perspective. The theme refers to how nurses expressed a need to support and strengthen parents to better promote healthy diet and activity behaviors in their children.

Nurses highlighted the need for–and importance of–targeting parents early and strengthening them in their parenting role, for instance by encouraging parents to take responsibility and set boundaries and routines for healthy behaviors. However, this was also described as a very sensitive and difficult issue to address as a health professional.

“It’s about daring to be the parent, and to set that framework [of clear boundaries], because you are the parent and you have the knowledge, and you therefore also need to take that responsibility; children cannot be burdened with it.” (Nurse No. 5, 3.5 years in the profession).

Avoiding conflict by not taking full responsibility for the foods served at home was a common issue described, where parents—according to the nurses—would often use the child’s feelings as an excuse for serving comfort foods. One nurse talked about the value of parents learning to say “no” and how that was difficult for many parents, i.e., to act more on the basis of long-term health consequences.

“What we perceive is common, is that ‘The children want to have this’. The child wants it [sweets, snacks]. And therefore, it is available at home […]. Saying no can also be an act of love.” (Nurse No. 10, 15 years in the profession).

Nurses did not perceive parents as worried or concerned about their child’s physical activity; instead, it was their diet that engaged them the most. Nurses also described how parents worried about their child eating too little rather than eating too much. This was more frequently observed among parents with ethnic- and socio-cultural backgrounds other than Swedish. This concern was also sometimes further strengthened by messages from Swedish primary child health care, which were perceived as conflicting by many parents. As one nurse expressed it, during the first year of life, the focus is on the young child growing steadily. Some parents continue with that idea after the age of one, and often feel confused when told that growth should slow down at two or three years of age. This concern was also linked to limited knowledge about portion sizes.

“Nowadays, people are very aware of sweets. But the amount of food or what they eat is not always focused on as much.” (Nurse No. 7, 8 years in the profession).

According to the nurses, despite having the best intentions, many parents serve their child large portion sizes, resulting in feelings of anxiety when the child is not able to finish the meal. Subsequently, parents end up using various tricks to get their child to eat up.

### 3.2. Preferences of Content and Features

The two final themes correspond to our third aim regarding useful and supporting content and features in an app targeting parents. Both parents and nurses suggested a variety of features and content. Depending on their needs, the parents’ preferences sometimes differed. However, there was also a lot of common ground. Child health care nurses talked about the relevance of a shared platform with clear information and pictures to show the parents, available in different languages.

#### 3.2.1. Interactive and Tailored Support

The parents described several features which they thought would be important in an app aiming to support them with healthy lifestyle behaviors, such as: comprehensive content, interactive functions and tailored support.

The data showed a need for a comprehensive app that would include social and emotional support rather than recommendations for healthy living per se. For example, one parent in the Arabic focus group talked about how overwhelming the experience of being a new parent could sometimes be and suggested adding content specifically addressing parental mental health and wellbeing.

“I need something for me as a mother […] from the point my child is one day old. And then for me as a mother of two children […] I’m a new mother again […]. Something for me, not just for the child, about all the emotions and everything that you go through.” (Mother No. 2, Arabic focus group, one child).

For the app to be comprehensive, this also meant including both general health advice and specific tips. For instance, parents from the Somali focus group expressed a need for their children to become better acquainted with traditional Swedish foods and suggested adding Swedish recipes to the app, for them to prepare and serve to their children a couple of times per week.

Common to all three focus groups were suggestions for the app to be interactive, using for instance online forums, or contact with a health care professional or a parent group. Parents in the Swedish focus group were also interested in an interactive support app, where they could choose for themselves to a certain extent which types of features and content to use.

Tailoring the content and structure of the app was also expressed as important. For example, parents suggested adding age-appropriate activities that would follow their child’s development from birth onwards, including tips on healthy recipes and snacks, and examples of healthy versus unhealthy foods. Including information about dental care for young children was also suggested. Tailoring was also about making the app available in several languages and finding the most appropriate delivery modes (such as text and audio/video). For example, for the parents in the Somali focus group it was essential for the app content to be available in Somali—both in text format, but most importantly in audio/video format, due to them having very few years of schooling in their home country, resulting in limited reading and writing skills.

In the Swedish focus group, tailoring was discussed in terms of including information about how to eat seasonally and recipes tailored for different types of families such as vegetarians, vegans or families were the child had an allergy. Parents in the Arabic focus group believed that it would be beneficial to add information about the long-term health consequences of serving unhealthy foods, as this would motivate them to make healthier choices for their children. They also suggested adding pictures and videos of children eating vegetables, to show their child, in order to reinforce their parental instructions.

The data showed differences in preferences regarding whether the app should remind parents with push notifications or just be available for users when needed. For example, common for both Arabic- and Somali- speaking parents was for the app to motivate and remind them to stay on track with healthy routines, by sending out feedback and reminders. However, in the Swedish-speaking focus group, parents did not like the idea of receiving push notifications.

“Good to have an app that keeps track of your lifestyle habits, such as sleep, have I achieved my goals? Or ‘Is this how it’s supposed to be?’ Then, it will also be on my agenda, like a reminder to me.” (Mother No. 4, Arabic focus group, one child).

“But I’m thinking that… the app shouldn’t reach out to me, instead I should be the one reaching for the app […]. When I feel I need the app, then I turn to it.” (Mother No. 5, Swedish focus group, three children).

#### 3.2.2. Need for a Shared Platform

Nurses described how a digital tool that parents and nurses could use together, would be valuable. The tool would serve as a shared platform with evidence-based information to be used when approaching sensitive topics such as child overweight.

“It would be both a support for me and a support for them, that we are actually doing this together.” (Nurse No. 3, 13 years in the profession).

The use of pictures was also expressed as something that would facilitate communication between parents and nurses. For example, pictures of age-appropriate portion sizes or different types of breakfast cereal could enable communication with parents speaking other languages and parents who had difficulty reading. Furthermore, nurses talked about how important it was for such a digital tool to be accessible for them, so that they also could monitor the parents’ progress and give tailored support.

“It’s important to use pictures, it should be as uncomplicated as possible […] not everyone reads well.” (Nurse No. 13, 9 years in the profession).

“You may still have to give some kind of feedback now and then […] you could meet with them [the parents] again after a couple of months and give positive feedback […] or you could have a phone conversation, hear how it is going and what their experience is like […]. You must have the personal contact as well.” (Nurse No. 9, 13 years in the profession).

Another use for a shared tool could be parents documenting the child’s food intake before a visit to the child health care center, simply by taking a picture of the food and drinks served during a regular day. As nurses often struggle to understand how food intake looks in different families, this would be an easy way of assessing that. Nurses also talked about how parents sometimes struggled to see the impact of diet on dental health and adding information about this was therefore suggested.

The data also showed that a shared tool, translated into several languages, could facilitate communication between nurses and immigrant families. According to the nurses, failing to reach parents with information about health behaviors during visits to the child health care center was quite common, and was often due to a combination of reasons. The family’s overall socioeconomic situation was a strong influencing factor, and the nurses described the most socioeconomically vulnerable as being the hardest to reach. Failure to reach parents was, however, first and foremost linked to linguistic barriers, which in turn, were more frequently connected to sociocultural differences in terms of eating habits and child feeding practices, such as serving larger portion sizes and worrying more about their children not eating enough.

“If there is something you bring with you, I believe, it’s your eating habits. There are cultural differences there, and it would be possible to reach through [to the parents]. And then you need it [informative material] in another language.” (Nurse No. 9, 13 years in the profession).

## 4. Discussion

### 4.1. Principal Findings

This study set out to explore needs and concerns among Somali-, Arabic-, and Swedish-speaking parents in terms of supporting healthy diet and activity behaviors in their children as well as nurses’ perceptions of parental needs and concerns in relation to this. The study also aimed to explore how the features and content of the MINISTOP 1.0 app could be refined to better support health behaviors in children, among both parents and nurses. The main findings identified several needs and challenges relating to supporting health behaviors in children and highlighted the importance of interventions targeting parents early to support and strengthen them in their parenting role. Furthermore, findings suggested that app features such as comprehensive content, interactive functions, and tailored support may be useful. Finally, the need to tailor mHealth interventions to different languages and cultures was underlined.

### 4.2. Comparison with Prior Work

The findings identified language as a barrier to communication between parents and nurses, and to accessing health information. Previous research indicates low levels of literacy in Somalia [31]. This was also prevalent in our findings, where the Somali mothers requested alternative modes of delivery (i.e., audio, video) for the app content. According to Mårtensson et al., health literacy among Somali and Arabic-speaking refugees in Sweden could be improved if health information were to be disseminated in native languages, within accessible channels [32]. Health literacy addresses the ability of an individual to obtain, process and understand the health information needed to make informed health decisions [33,34]. It also addresses the ability of health care services to meet the diverse needs of multi-cultural populations [33,34]. Gele et al. reported on the adverse effects of low literacy levels on health literacy and health outcomes among Somali immigrants in Norway, further strengthening the importance of adapting the level of dissemination for health information [35]. Thus, translation alone would not be sufficient to reach all parents interested in using such an app.

Nurses mentioned how parental concerns regarding children eating too little were common, and more frequently observed among parents with an immigrant background. Nurses also struggled to reach through to parents regarding this, especially when there were also linguistic barriers. This was in line with the views of the Somali mothers, who were concerned about their children’s food intake being inadequate, and nurses further connected this concern to limited knowledge about portion sizes for young children. Similarly, Arcan et al. found that Somali parents in the US were interested in interventions tailored to their culture, including information about portion sizes [17]. Previous research also indicates that higher rates of infant overweight are connected to maternal beliefs about infant weight and long-term health [18,36,37]. It is important to acknowledge the impact of underlying structural, social, and environmental factors on the preservation of such beliefs [16,38,39]. In the current study, nurses talked about how they connected overweight and obesity in children with large serving sizes from a very young age, rather than due to excessive intake of sweets and snacks. This indicates a need for parental interventions offering guidance on portion sizes to ease parental concerns, as well as a preventive measure against childhood overweight and obesity.

Another interesting finding was the diverse perceptions between parents in the Swedish and Arabic focus groups regarding health behavior recommendations. In the Swedish focus group, parents discussed the stresses of trying to adhere to dietary recommendations, and how that stress sometimes generated feelings of failure. This was in agreement with findings from Ljungkrona-Falk et al., where Swedish child health care nurses talked about the stressors of modern society and how these hindered parents in supporting healthier behaviors in their children [40]. By contrast, Arabic-speaking parents requested information about both recommendations and the long-term health consequences if these were not met. These findings underline the importance of framing health information in a way that is sensitive and understanding of the problems many parents face in their everyday lives, and of providing attainable strategies and solutions for reaching these.

The findings indicated that physical activity was not a major concern among parents. Likewise, Regber et al. found that Swedish child health care nurses perceived parents to be confident about their child’s level of activity at preschool [41], while a study on school-aged children in a multi-ethnic community in Sweden found parents to be more concerned about their children’s diet rather than their physical activity [42]. The two- to three-year age range is a period where children become increasingly mobile and explore their surroundings, often demonstrated by an intermittent movement pattern [43]. This may also explain why parents worry less about physical activity during this developmental stage. Regardless, accumulated research on older children shows how difficult it is to adhere to physical activity guidelines [44,45]. More specifically, only two out of ten school-aged children in Sweden meet the recommended daily amount of physical activity [46]. A recent pilot study on Swedish four-year-old children showed that only 19% met the 2019 WHO guidelines on physical activity, sedentary behavior and sleep for children aged 0–5 years [47]. The main driver of this finding was excessive screen time, and parents raised challenges in limiting screen time even at this age. Hence, even though many parents do not consider physical activity to be a major problem at this young age, promotion of healthy movement behaviors including strategies to handle screen time is a key feature needed in future interventions.

### 4.3. Strengths and Limitations

A strength of this study was using two different data sources (parents and nurses). Focus groups [48] were used to collect group level data and data from person-to-person interactions among the parents. Further, the group discussions facilitated genuine discussions on the content of the app as well as suggestions for new content and features. Individual interviews [25] were conducted as it was not feasible to bring together nurses from centers throughout Sweden. However, telephone interviews with nurses from different socioeconomic and geographic areas provided diverse experiences. In addition, studies have shown that there is little or no difference in the quality of the data collected over the phone compared to face-to-face [49].

According to qualitative methodology, data saturation should be consistent with the aim of the research [25,30]. Specifically, the focus of inductive thematic saturation is to identify new codes and themes in the data [30]. In our study, thematic saturation was reached when no new codes or themes were found. When combined, data richness from the focus group interviews and individual interviews was sufficient to answer our research questions. Purposeful sampling and recruitment through Somali- and Arabic-speaking bridge-builders was used in an area with a predominantly immigrant population to ensure the participation of parents who spoke Somali and Arabic. The bridge-builders were a strength of our study and were especially important for the verbal exchange with the parents, i.e., for explaining the interview aims and procedure. For the individual interviews, convenience sampling was used to recruit child health care nurses from centers that had agreed to participate in the MINISTOP 2.0 trial [24] across Sweden. Potential limitations include the number of focus groups, which highlights the need for caution when interpreting the results. Nevertheless, the group interviews provided several ideas about what parents may want and need in a support app, and these contributed to the adaptations of the app. Moreover, the good representation of primary child health care centers in Sweden and the varying socioeconomic position and ethnicity of the parents supports trustworthiness in terms of transferability of the results [50].

Furthermore, several steps were taken to increase the trustworthiness of the study. Credibility was endorsed in data collection and analysis primarily through investigator triangulation. Specifically, involving researchers with various backgrounds in nutrition, rehabilitation and behavioral science, including the development and evaluation of mHealth interventions, as well as qualitative research methods, can have increased the credibility of the findings [50]. In addition, two authors (CA and UM) independently coded the transcripts to further improve the credibility of the findings. Dependability was enhanced by employing rigorous and systematic procedures in data collection and analysis in accordance with the steps described by Braun and Clarke [28,29], increasing the consistency of the findings. Finally, the use of quotations to illustrate the findings can enhance trustworthiness in terms of transparency and thus increase the applicability of the findings [50]. The Consolidated Criteria for Reporting Qualitative Research (COREQ) 32-item checklist [26] was used to logically report our approaches throughout the study.

### 4.4. Implications

Major findings from this study include linguistic barriers in parent-nurse communication, parental concerns about their children eating too little, limited knowledge about portion sizes for young children, and diverse views on diet and physical activity recommendations. These findings provided valuable insights that were used to modify and improve the MINISTOP 1.0 app into the MINISTOP 2.0 version. Thus, after translation of the app into Somali, Arabic and English, we added audio-video files of the text content in Somali and Arabic. To support and reassure parents about their children’s eating behaviors and food intake, we added videos on the importance of allowing children to self-regulate their food intake as well as a range of parental practices supporting this. Pictures of portion sizes for children aged two to three years were also added, to further visualize the average amount of food required during a meal for children of that age. Furthermore, pictures illustrating daily recommendations for fruit and vegetable intake, and the maximum weekly intake of sweets, savory snacks and sweetened beverages were added, as were tips and strategies to make these recommendations more attainable. To inspire parents to increase their children’s physical activity, we included short videos of simple and fun outdoor and indoor activities for preschool-aged children. Due to budget and time constraints, we were not able to act on all suggestions from the participants. However, we are considering adding some of these in future versions of the app, such as a feature for uploading pictures of meals for the nurse to look at, and access to an online parental forum via the app. In response to the diverse views on the app sending out reminders and messages in the form of push-notifications we added an introductory video explaining how parents can use the content and features of the app to best fit their personal everyday life and needs. Furthermore, push notifications can also be turned off. Finally, for Version 2.0 of the MINISTOP app, we collaborated with the Swedish National Dental Health Agency to create a comprehensive theme on dental health for preschool-aged children. Thus, besides being an evidence-based parental support tool that can be scaled up at a low cost, the MINISTOP 2.0 app is now also tailored to be inclusive of the population of Somali- and Arabic-speaking parents in Sweden, regardless of literacy level. Therefore, the app has the potential to assist primary child health care nurses in their work to promote health behaviors in children, facilitate their communication with parents speaking other languages, and help decrease health inequalities early in life.

## 5. Conclusions

This study contributes valuable insights and knowledge about challenges and needs of parents in terms of supporting health behaviors in their children, as well as relevant adaptations in a parental support app promoting this. Early access to information and strategies to strengthen parents in their role was highlighted. In addition to translation, findings from both parents and nurses motivated addition of audio/video files of the content to make the app more accessible. Findings also motivated addition of content addressing parental concerns about children’s diet and eating behaviors. Parents reported less concern with their children’s physical activity level, which was confirmed by the nurses. This adapted version of the app—MINISTOP 2.0, has the potential to be useful for childhood obesity prevention in multi-ethnic communities.

## Figures and Tables

**Table 1:** Characteristics of participating parents * and child health care nurses.

	Somali Focus Group (*n* = 5)	Arabic Focus Group (*n* = 4)	Swedish Focus Group (*n* = 6)
	Mean ± SD ^1^	Mean ± SD	Mean ± SD
Age (years)	34.0 ± 6.6	31.2 ± 2.0	35.8 ± 4.7
Education (years)	5.5 ± 3.3	13.5 ± 1.7	14.5 ± 0.5
Years in Sweden	9.8 ± 5.3	8.7 ± 4.6	
Number of children	5.2 ± 2.2	1.3 ± 0.5	1.3 ± 0.8
Enrolled with one or more children in Swedish daycare (years)	6.8 ± 5.4	1.3 ± 0.3	2.1 ± 1.1
	**Child health care nurses (*n* = 15)**		
	**Mean ± SD**		
Age (years)	46.9 ± 8.2		
Years in the profession	7.5 ± 3.9		

* All parents in the Swedish- and Somali-speaking focus groups were born in Sweden and Somalia respectively. In the Arabic-speaking focus group, three of the parents were born in Iraq, and one parent in Syria. ^1^ SD, standard deviation.

**Table 2:** Study aims and corresponding themes, representing both the parents’ and nurses’ perspectives.

Aim 1:	Aim 2:	Aim 3:	
Needs and concerns among parents	Nurses’ perceptions of parental needs and concerns	Preferences of features and content in the app	
**Theme:**	**Theme:**	**Theme:**	**Theme:**
Parental challenges	Supporting parents	Interactive and tailored support	Need for a shared platform

## Supplementary Materials

The following are available online at https://www.mdpi.com/article/10.3390/nu13072190/s1, Figure S1: Consolidated Criteria for Reporting Qualitative research (COREQ) checklist; Table S1: interview questions for Somali-, Arabic-, and Swedish-speaking parents; Table S2: interview questions for Swedish child health care nurses.
