# Supplementary material for: Adapting a Parental Support App to Promote Healthy Diet and Physical Activity Behaviors (MINISTOP) for a Multi-Ethnic Setting: A Qualitative Study on the Needs and Preferences of Parents and Nurses within Swedish Child Health Care
Source: Nutrients. 2021 Jun 25;13(7):2190. doi: 10.3390/nu13072190 (PMC8308428; doi:10.3390/nu13072190)
Supplement: Supplementary file 1 [file nutrients-13-02190-s001.zip › nutrients-1194707-supplementary.pdf]

nutrients

Supplementary

Adapting a Parental Support App to Promote Healthy Diet and Physical Activity Behaviors (MINISTOP) for a Multi-Ethnic Setting: A Qualitative Study on the Needs and Preferences of Parents and Nurses within Swedish Child Health Care

Christina Alexandrou [1,2,*], Ulrika Müssener [1], Kristin Thomas [1], Hanna Henriksson [1] and Marie Löf [1,2]

Topic	Item No.	Guide Questions/Description	Reported on Page No.
**Domain 1: Research Team and Reflexivity**			
Personal Characteristics			
Interview/Facilitator	1	Which author/s conducted the interview or focus group?	4
Credentials	2	What were the researcher's credentials? (e.g. PhD, MD)	3, 4
Occupation	3	What was their occupation at the time of the study?	3, 4
Gender	4	Was the researcher male or female?	3, 4
Experience and Training	5	What experience or training did the researcher have?	3, 4
Relationship with Participants			
Relationship Established	6	Was a relationship established prior to study commencement?	3, 4
Participant Knowledge of the Interview	7	What did the participants know about the researcher? (e.g. personal goal, reasons for doing the research)	3, 4
Interviewer Characteristics	8	What characteristics were reported about the inter viewer/facilitator? (e.g. bias, assumptions, reasons and interests in the research topic)	4
**Domain 2: Study Design**			
Theoretical Framework			
Methodological Orientation and Theory	9	What methodological orientation was stated to underpin the study? (e.g. grounded theory, discourse analysis, ethnography, phenomenology, content analysis	4
Participant Selection			
Sampling	10	How were participants selected? (e.g. purposive, convenience, consecutive, snowball)	3, 10, 11
Method of Approach	11	How were participants approached? (e.g. face-to-face, telephone, mail, email)	3
Sample Size	12	How many participants were in the study?	3, 5
Non-participation	13	How many people refused to participants or dropped out? Reasons?	3
Setting			
Setting of Data Collection	14	Where was the data collected? (e.g. home, clinic, workplace)	3, 4
Presence of Non-participants	15	Was anyone else present besides the participants and researchers?	4
Description of Sample	16	What are the important characteristics of the sample? (e.g. demographic data, date)	5
Data Collection			
Interview Guide	17	Were questions, prompts, guides provided by the authors? Was it polit tested?	3, 4, 11
Repeat Interview	18	Were repeat inter views carried out? If yes, how many?	NA
Audio/Visual Recording	19	Did the research use audio or visual recording to collect the data?	3, 4
Field Notes	20	Were field notes made during and/or after the inter views or focus group?	4
Duration	21	What was the duration of the inter views or focus group	4

Data Saturation	22	Was data saturation discussed?	4, 10
Transcripts Returned	23	Were transcripts returned to participants for comment and/or collection?	NA
**Domain 3: Analysis and Findings**			
Data Analysis			
Number of Data Codes	24	How many data coders coded the data?	4, 11
Description of the Coding Tree	25	Did authors provide a description of the coding tree?	NA
Derivation of Themes	26	Where themes identified in advance or derived from the data?	4
Software	27	What software, if applicable, was used to manage the data?	NA
Participant Checking	28	Did participants provide feedback on the findings?	NA
Reporting			
Quotations Presented	29	Were participant quotations presented to illustrate the themes/findings? Was each quotation identified? (e.g. participant number)	4–9
Data and Findings Consistent	30	Was there consistency between the data presented and the findings?	4–9
Clarity of Major Themes	31	Were major themes clearly presented in the findings?	4, 5
Clarity of Minor Themes	32	Is there a description of diverse cases or discussion of minor themes?	4–9

Developed from: Tong, A.; Sainsbury, P.; Craig, J. Consolidated criteria for reporting qualitative research (COREQ): a 32-item checklist for interviews and focus groups. *International Journal for Quality in Health Care* **2007**, *19*, 349–357.

Once you have completed this checklist, please save a copy and upload it as part of your submission. DO NOT include this checklist as part of the main manuscript document. It must be uploaded as a separate file.

Figure S1. COREQ (COnsolidated criteria for REporting Qualitative research) Checklist. A checklist of items that should be included in reports of qualitative research. You must report the page number in your manuscript where you consider each of the items listed in this checklist. If you have not included this information, either revise your manuscript accordingly before submitting or note NA.

Table S1. Interview questions for Somali-, Arabic-, and Swedish-speaking parents.

**Interview questions to parents regarding their need of an app to support healthy diet and physical activity behaviors in their children:**	
1.	What are your opinions/views on receiving information about multiple health behaviors at the same time (i.e., diet, physical activity and screen time)?
2.	What type of information and support would you as parents need to support healthy diet and activity behaviors in your child? For example, what type of support would you need in order to make changes in your everyday routines, to make these healthier?
3.	We have developed an app to help parents support a healthy diet and regular physical activity in their children. Among other things, we have included a registration and feedback feature. What are your thoughts about such a feature?
4.	What would you like to register and receive feedback on?
5.	How frequently would you register your child's behaviors?
6.	We are thinking about sending out information in the form of push-notifications in the app. What are your thoughts/opinions about that?
7.	What type of information/tips would you like to receive in these push-notifications?
8.	How frequently would you like to receive push-notifications?
9.	What do you think about including videos with tips and ideas in the app; what would you like to know more about/see in such a video?
**Interview questions regarding the features and content of the MINISTOP 1.0 app. Parents are shown screenshots of the app features and content to be able to reflect on these:**	
10.	What do you think about the app now that you have seen it (layout/design and content)? Was there anything you didn't like or thought looked strange?
11.	Is there anything missing? Information or features?
12.	We were thinking about including parents of children aged 2–3 years to test the app in a study. What are your thoughts about receiving such an app at that time period in your child's development?
13.	What would it take for you to use such an app? (e.g., what would be crucial/important to include?)
14.	Have you ever used or are you using a similar app today? If yes: Which one? What features does that app include? Is there anything you miss in that app?
15.	Is there anything else you believe is important for us to keep in mind when we develop the app further?

Table S2. Interview questions for Swedish primary child health care nurses.

**Interview questions for Swedish primary child health care nurses about current working routines, parental challenges in terms of supporting their children's health behaviors and the use of a parent-oriented mHealth solution in practice:**	
1.	What are your current health practice routines within primary child health care, in terms of healthy diet and activity behaviors, to prevent future adverse health outcomes in children in general?
2.	What are your current practice routines for treatment of children with overweight or obesity?
3.	What problems or difficulties do you experience/encounter with the current way of working, if you first think about it generally and preventively?
4.	If you think about it in terms of obesity treatment, what problems or difficulties do you experience with the current way of working?
5.	What do you find is most challenging for families/parents when it comes to healthy eating behaviors, physical activity and screen time?
6.	Do you experience any differences in challenges between families/parents that have children with overweight or obesity?
7.	What are your experiences/perceptions of reaching families in need of support in general, with information on health behaviors for children?
8.	What are your experiences/perceptions of reaching families where the child has obesity, in terms of health behaviors?
9.	As I mentioned earlier, we want to explore how to best support health behaviors in children through a smartphone app. In general, what advantages/disadvantages do you see with using such an app for prevention?
10.	Could you see any advantages/disadvantages with using such an app for childhood obesity treatment?
11	Are there any benefits with using such an app for general prevention, compared to current working methods?<br>a) For the families?<br>b) For you as a health care professional?<br>c) If you think about it in terms of childhood obesity treatment, are there any benefits compared to current working/practice methods?<br>d) Are there any specific (population) groups that you believe could benefit more from extra support through an app?
12.	If you think in terms of prevention, how do you think it would work to use such an app?
13.	How do you think it would work to use such an app, if you think in terms of childhood obesity treatment?
14.	If you were to recommend an app like this to families, what type of content and/or features do you think the app should include, in order for you to be willing to recommend it for prevention?
15.	If you think in terms of obesity treatment, what would be important for the app to include in order for you to be willing to recommend it to families?
16.	What would need to change/is there anything that needs to change in your current health practice routines, for you and your colleagues to be able to use the app, if you think both in terms of prevention and in terms of obesity treatment? Would it be difficult for you to use the app, or could you see the app as a complement?
**Interview questions regarding the features and content of the MINISTOP 1.0 app. The nurse is shown screenshots of the app features and content to be able to reflect on these:**	
17.	Overall, what do you think about the content in the app? (both in terms of prevention and obesity treatment)
18.	Are the 12 different themes in the app relevant to the target group (parents with children aged 2-3 years)?
19.	Are there any themes that you are missing and would like us to add?
20.	Is there anything else missing?
21.	What is your opinion on families receiving information about several health behaviors (i.e., diet, physical activity and screen time) at the same time in an app?
22.	Do you use any other app in your work/practice? How does that work?
23.	Is there anything else we should think about when developing the app further?
